# Examining fronto-limbic brain and sleep mechanisms of antidepressant effects in cognitive-behavioral therapy for insomnia

**DOI:** 10.1038/s41386-026-02431-0

**Published:** 2026-05-07

**Authors:** Adam J. Krause, Raquel Osorno, Natalie L. Solomon, Maryam Ahmadi, Pandora Lam, Olivia Magana, Emilija Blozyte-Sakenis, Leah N. Harris, Madeline C. Babros, Sarah S. Izabel, Rebecca A. Bernert, Leanne M. Williams, James J. Gross, Jun Ma, Laura C. Lazzeroni, Jerome A. Yesavage, Rachel Manber, Jared M. Saletin, Andrea N. Goldstein-Piekarski

**Affiliations:** 1 Department of Psychiatry and Behavioral Sciences, Stanford University, Stanford, CA USA; 2 Mental Illness Research, Education and Clinical Center, VA Palo Alto Health Care System, Palo Alto, CA USA; 3 Department of Psychology, Stanford University, Stanford, CA USA; 4 Department of Medicine, University of Illinois Chicago, Chicago, IL USA; 5 Department of Psychiatry and Human Behavior, Brown University, Providence, RI USA; 6 Center for Sleep and Circadian Rhythms in Child and Adolescent Mental Health, Emma Pendleton Bradley Hospital, East Providence, RI USA

**Keywords:** Depression, Amygdala, Sleep

## Abstract

Treating insomnia with Cognitive-Behavioral Therapy for Insomnia (CBT-I) improves depression symptoms, but the underlying mechanisms remain unknown. This single-arm mechanistic trial (ClinicalTrials.gov, NCT04424407) examined fronto-limbic and sleep mechanisms of CBT-I’s antidepressant response in 48 participants (64% female; age 25–60) with insomnia and depression symptoms. Participants completed functional magnetic resonance imaging (fMRI), polysomnography (PSG), and symptom assessments before and after 6 CBT-I sessions. CBT-I resulted in reduced amygdala reactivity to fearful faces (*d* = 0.55, *p* = 0.008). Depression and sleep (objective and self-reported insomnia symptoms also improved. However, fMRI-assessed fronto-limbic changes were not associated with a reduction of depressive symptom severity. Instead, reduced depressive symptoms correlated with reduced self-reported insomnia symptoms (*p* = 0.001, *η*^2^*p* = 0.19) and increased objective sleep efficiency (*p* = 0.04, *η*^2^*p* = 0.10). Notably, pre-treatment PSG-assessed sleep efficiency, but not fronto-limbic function nor insomnia symptoms, predicted reduced depressive symptoms (*p* = 0.007, *η*^2^*p* = 0.16), suggesting that lower objective sleep efficiency prior to treatment may be associated with greater antidepressant benefit from CBT-I.

## Introduction

Depression and insomnia disorders are both highly prevalent and debilitating conditions that frequently co-occur [1, 2]. Insomnia is a diagnostic feature of depression, a major risk factor for its development, and can exacerbate depressive symptom severity [1, 3]. Critically, insomnia increases suicide risk across the lifespan even when accounting for other symptoms [3–9], pointing to insomnia as a promising transdiagnostic target for alleviating depressive symptoms beyond sleep disturbances [10].

This overlap points toward shared underlying neurobiological mechanisms. Sleep problems may contribute to depression [11] by altering brain networks implicated in emotional processing and regulation. One promising neurobiological mechanism is fronto-limbic brain function. Emotional processing involves two parallel pathways [12, 13]: a direct, automatic pathway involving rapid bottom-up reactivity to emotional stimuli, and an explicit pathway involving conscious appraisal and contextual processing. The amygdala is at the core of both pathways. In the direct, automatic pathway, the amygdala works in concert with more ventral regions of the medial prefrontal cortex (mPFC), including the cingulate, to rapidly detect and respond to threat signals. In the explicit pathway, the amygdala engages with more dorsal regions of the mPFC during conscious emotional processing [14–22].

Neuroimaging studies can probe these parallel pathways using tasks designed to isolate automatic and explicit emotional processing. Nonconscious presentation of emotional stimuli isolates the automatic, bottom-up pathway [23], while conscious emotional tasks engage both bottom-up reactivity and explicit appraisal processes involving the mPFC [14–19]. The functional connectivity between the amygdala and mPFC regions reflects coordinated activity between these structures, suggesting modulatory interactions between these regions, consistent with top-down regulation.

Dysfunction in this fronto-limbic circuit has been observed in major depressive disorder (MDD) [24–36] and is related to poor emotion processing. Both amygdala hyper-reactivity and altered amygdala-mPFC connectivity are theorized to contribute to negative affective bias and threat sensitivity in some forms of depression [37–41].

These same emotion processing mechanisms are also altered by sleep disturbances. Most prior neuroimaging research using fMRI has examined experimental sleep deprivation or restriction paradigms that reduce sleep duration, often in healthy individuals. These studies show that experimental acute sleep deprivation and sleep restriction, and poor habitual self-reported sleep quality (including insomnia disorder) amplify amygdala reactivity to negative experiences [42–48]. For example, Motomura et al. [47] used chronic partial sleep restriction (4 h/night for 5 nights), which more closely approximates cumulative sleep debt than acute total deprivation, and found increased amygdala reactivity and decreased amygdala-cingulate connectivity associated with worse mood. Conversely, there is evidence that recovery sleep following sleep deprivation normalizes amygdala reactivity and re-establishes amygdala-mPFC connectivity [49]. Additionally, inter-individual differences in amygdala-mPFC connectivity following sleep deprivation correlate with concurrent anxiety increases [47].

However, clinical insomnia disorder differs importantly from experimental sleep deprivation. Insomnia is characterized by poor sleep despite adequate opportunity for sleep, and its diagnosis is based on subjective experience rather than objective sleep measurements. Moreover, not all individuals with insomnia show objectively short sleep duration.

Neuroimaging research specifically in insomnia populations remains very limited. Prather et al. [48] found that among poor sleepers, assessed using the Pittsburgh Sleep Quality Index questionnaire, depressive symptoms were associated with heightened amygdala reactivity, demonstrating that naturally occurring sleep disturbances relate to altered amygdala functioning. Baglioni et al. [50] reported increased amygdala reactivity to insomnia-related stimuli in individuals with insomnia disorder but without depression. While limited, this literature suggests that amygdala reactivity may be involved across several sleep disturbance profiles.

Critically, if sleep disturbance alters fronto-limbic function in ways that contribute to depression, this process may be reversible with targeted sleep intervention, such as cognitive-behavioral therapy for insomnia (CBT-I). CBT-I is the first-line non-pharmacological treatment for insomnia with established efficacy [51–54]. As recently outlined [55], controlled trials or longitudinal studies examining neuroimaging and objective sleep measurements together in treatment contexts are limited, particularly in clinical insomnia populations. The current two-phase trial addresses this gap by integrating multi-modal sleep and brain assessments to examine mechanisms of treatment response in CBT-I. This includes employing emotional processing tasks designed to probe automatic nonconscious reactivity, as well as explicit, conscious appraisal, allowing for identification of which specific fronto-limbic pathways are responsive to treatment.

Notably, CBT-I not only improves insomnia symptoms but also reduces comorbid depressive symptoms, including suicidal ideation [56, 57], but the mechanism remains unknown. This two-phased clinical trial tests whether impaired fronto-limbic brain function causally links insomnia and depression, specifically determining whether CBT-I improves depressive symptoms by reducing amygdala emotional reactivity and increasing amygdala-mPFC connectivity during emotional processing. Understanding whether CBT-I, the first-line intervention for insomnia disorder, operates directly through specific emotion processing pathways or indirectly through improved sleep can help provide mechanistic interpretability of how CBT-I reduces depressive symptoms.

The primary goal of the current report is to present results from the first phase of the two-phase clinical trial. This mechanistic, single-arm phase established whether CBT-I engages proposed fronto-limbic brain targets in individuals with comorbid insomnia and depression. We hypothesized that following CBT-I treatment, participants would experience reduced amygdala emotional reactivity and an increase in amygdala-mPFC functional connectivity, representing normalization of fronto-limbic emotional brain function.

We also hypothesized that CBT-I would be associated with improvements in both depression and sleep outcomes. Our primary clinical hypothesis was that CBT-I would reduce depressive symptom severity, with suicidal ideation as a secondary outcome. For sleep, we evaluated objective sleep efficiency as the primary outcome and self-reported insomnia severity as the secondary outcome. We selected polysomnography (PSG)-derived sleep efficiency as the primary objective outcome because sleep restriction is a component of CBT-I that aims to increase sleep efficiency, which is reduced in both insomnia and depression [58–60], and it is the most commonly used objective sleep quality measure [61, 62].

Building on these primary hypotheses, we also examined neural correlates of clinical improvement, testing whether the fronto-limbic normalization and sleep improvements were associated with reduced depressive symptoms. Additionally, we identified baseline predictors of antidepressant treatment response by examining whether pre-treatment differences in fronto-limbic function or sleep predicted which participants experienced the greatest antidepressant benefit from CBT-I.

To our knowledge, this is the first study examining a neurobiological model connecting depressive symptoms and sleep through their overlap in affective brain systems, elucidating the mechanisms by which CBT-I treatment improves depression and sleep.

## Methods

This manuscript presents primary results from the mechanistic clinical trial ClinicalTrials.gov NCT04424407 conducted at Stanford University. The overall trial aims to test the mechanisms of an established sleep intervention (CBT-I) in reducing depressive symptoms through improved fronto-limbic emotional brain function in individuals with elevated depressive symptoms and sleep disturbance. The current single-arm R61 phase primarily aims at establishing fronto-limbic target engagement. Target engagement is defined as the treatment effect reducing amygdala reactivity and increasing amygdala-mPFC connectivity during emotion reactivity and regulation paradigms. The study protocol was approved by the Institutional Review Board at Stanford.

### Participants

Participants were recruited from within 60 miles of Stanford University. All participants exhibited clinically meaningful insomnia and depression symptoms (Beck Depression Inventory, BDI≥14), operationalized as having complaints of sleep disturbance for at least 3 months (assessed during structured clinical interviews) but not at imminent risk for suicide, as assessed by the Columbia-Suicide Severity Rating Scale. The eligibility criterion for insomnia symptoms, initially set at an Insomnia Severity Index (ISI) score of ≥15, was revised to ≥10 to align with recent trials [63] and to reflect an optimal cutoff for detecting clinical insomnia [64]. Participants previously excluded solely for ISI scores of 10-14 were recontacted and reassessed for study enrollment, but none were subsequently enrolled due to not meeting other eligibility criteria or loss to follow-up. All participants were fluent and literate in English and provided written informed consent.

The study excluded participants according to the following: other sleep/circadian disorders, medications affecting sleep/alertness/mood, >14 alcoholic drinks/week or >4 drinks/occasion, medical diagnoses interfering with assessments, substance abuse/dependence, traumatic brain injury, severe sensory/motor impediments, pregnancy/breastfeeding, current/lifetime bipolar disorder/psychosis, current/expected psychotherapy for other conditions, CBT-I within past year, acute/unstable chronic illness, recent trauma exposure, rotating shift work, untreated moderate-severe sleep apnea (AHI ≥ 15). Forty-eight participants were included in the current analyses.

**Fig. 1:**
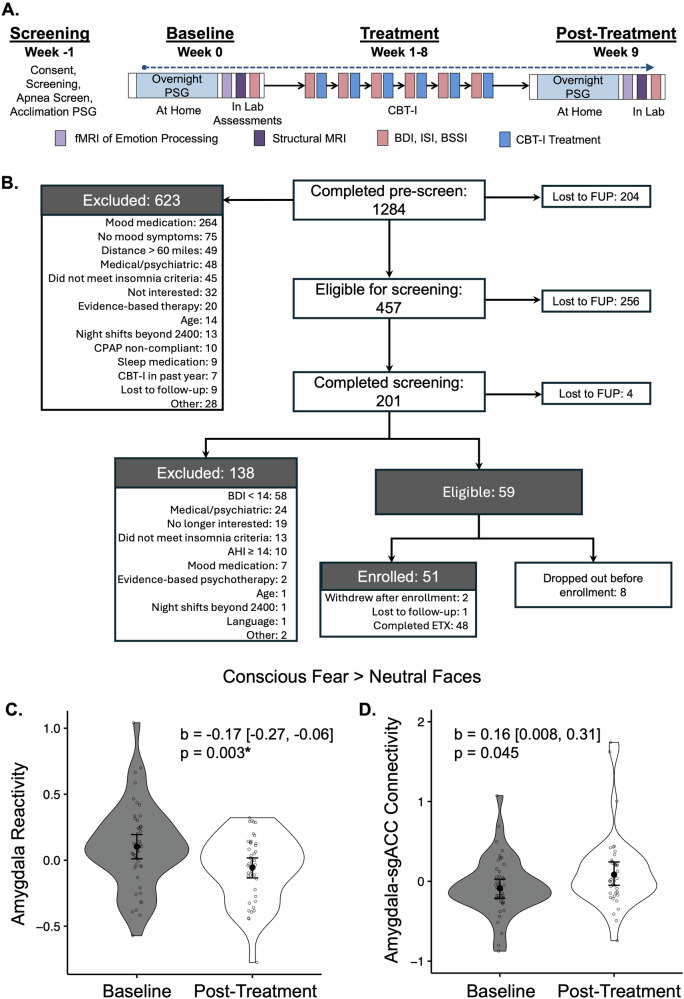
Study design, consort flow diagram, primary fronto-limbic outcomes. **A** Fifty-one participants were enrolled in the study, which included a screening session, pre-treatment baseline, a period of weekly therapist-guided CBT-I treatment, and a post-treatment session. Overnight PSG was collected before and after CBT-I treatment using at-home ambulatory recordings, followed by fMRI scanning and clinical outcomes assessments the next morning in the laboratory. **B** CONSORT flow diagram of participant progression through the trial. The primary analysis was based on an intention-to-treat principle and included all 51 participants who completed pre-treatment baseline, and 48 participants completed the study in its entirety. Pre-treatment baseline and post-treatment values for **C** amygdala reactivity and **D** subgenual anterior cingulate cortex (sgACC) connectivity when viewing fearful relative to neutral faces in the conscious condition of the Faces task. Regression coefficients [95% CI] are from linear mixed-effect models. P-values are uncorrected, and the asterisk (*) marks significance following FDR-correction. Error bars represent 95% confidence intervals. PSG, polysomnography; CBT-I, cognitive-behavioral therapy for insomnia; fMRI, functional magnetic resonance imaging; BDI, Beck Depression Inventory; ISI, Insomnia Severity Index; BSSI, Beck Scale for Suicide Ideation; AHI, apnea-hypopnea index; CPAP, continuous positive airway pressure; FUP, follow-up; ETX, end of treatment.

Prospective participants underwent two-stage screening: remote video calls with consent, questionnaires, and clinical interviews assessing sleep and mood, followed by in-person screening with overnight at-home ambulatory PSG (Compumedics Siesta 802, BrainVision Easycap) and sleep apnea screening (ResMed Apnealink Air). Participants completed three overnight ambulatory PSG recordings at screening/habituation, pre-treatment baseline, and post-treatment (Fig. 1A). At baseline and post-treatment, participants completed overnight PSG, symptom questionnaires, and fMRI scans the following morning. MRI scans were collected at identical times at each timepoint, 3 h post-wake, to control circadian and sleep inertia confounds. Daily sleep diaries confirmed CBT-I adherence, and actigraphy (MicroMotionlogger) corroborated sleep timing.

### Insomnia treatment

Participants received six sessions of therapist-delivered CBT-I over 8 weeks. CBT-I is the gold-standard treatment for insomnia [65], utilizing behavioral therapy (sleep restriction and stimulus control), cognitive therapy addressing maladaptive sleep-related thoughts, and sleep education. Daily sleep diaries were used for time-in-bed adjustments and determining PSG sleep period times.

### Study outcomes

#### Fronto-limbic brain function

Primary outcomes of fronto-limbic emotional brain function include amygdala reactivity as well as amygdala-mPFC connectivity, as measured by psychophysiological interaction [66] (PPI), assessed using validated neuroimaging tasks of emotional reactivity and regulation. We focused on the amygdala and mPFC due to prior findings that they are sensitive to sleep status and are altered in depression. Cortical ROIs were defined a priori [13] (see Supplement). Briefly, clinically-relevant circuits were generated through a meta-analytic search using Neurosynth [67] (RRID:SCR_006798) to create a “Negative Affect” uniformity map. Five regions of the mPFC were selected for the current analyses, including the dorsal anterior cingulate (dACC), ventromedial prefrontal (vmPFC), dorsomedial prefrontal (dmPFC), subgenual anterior cingulate (sgACC), and pregenual anterior cingulate (pACC) cortices. The sgACC did not meet quality control metrics for temporal signal-to-noise ratio, but given the difficulty of imaging this region, its importance to defining the negative affect circuit, and prior imaging findings in depression, we included the region in the current analyses. For the subcortical amygdala, an anatomical definition from the AAL atlas was used [68].

### Depression and sleep

The Beck Depression Inventory-II [69] total score, excluding the sleep item, was the primary outcome measure of depression symptoms, assessed at pre-treatment baseline, weekly before treatment sessions, and post-treatment. The BDI is a 21-item self-report scale assessing depression severity. The ‘changes in sleeping pattern’ item was removed to assess changes in depression symptoms independently from the established CBT-I efficacy on sleep symptoms [56, 70].

The Beck Scale of Suicidal Ideation was the secondary outcome of emotional distress. The BSSI is a 21-question evaluation measuring a broad spectrum of attitudes and behaviors related to suicide [71].

The primary objective sleep outcome was PSG-assessed sleep efficiency (percent of total sleep opportunity spent asleep), measured pre- and post-treatment.

Self-reported insomnia symptoms were measured using the ISI total score [72], administered at pre-treatment baseline, weekly before treatment sessions, and post-treatment.

### Imaging tasks

The Facial Expressions of Emotion Task reliably activates the amygdala [12, 22, 29, 73, 74], described fully in Korgaonkar et al. [75] (see Supplement), with two conditions: conscious (500 ms presentation) and nonconscious (16.7 ms presentation with neutral face mask) [22]. The Emotion Regulation Scenes Task asked participants to “look” or “decrease” their emotional response to negative or neutral valence images from the International Affective Picture System [76], described fully in Fonzo et al. [77] and Minkel et al. [78].

### Polysomnography

PSG was used to measure sleep using a 32-channel system (see Supplement). Sleep staging was performed in accordance with standardized techniques [79].

### Sample size

We initially targeted enrollment of 70 participants. However, due to recruitment-related challenges arising from the COVID-19 pandemic, this enrollment target was adjusted to at least 50 participants. The final sample size of 51 enrolled participants provides 80% power to detect treatment effects on fronto-limbic activation and connectivity of Cohen’s d = 0.40 (small to medium effect sizes) at Type 1 error *α*=0.05.

### Statistical analyses

We conducted a modified intent-to-treat analysis, including all data unless artifactual or corrupted. The primary analysis examined treatment-associated changes in amygdala reactivity across tasks with FDR correction applied for each task [80]. For tasks/contrasts showing significant amygdala reactivity reductions, we tested whether these corresponded to parallel increases in mPFC connectivity. We applied linear mixed-effects (LME) models with random intercepts at the participant level, including age and sex as covariates.

Treatment-related clinical changes were tested using LME models with random intercepts. Given the zero-inflation in suicidal thinking (BSSI), we used generalized LME models for the negative binomial family.

Neural correlates of clinical improvement were examined using linear regression with change scores (post minus pre-treatment). For models with BSSI outcomes where residuals violated normality (Shapiro-Wilk test, *p* < 0.05), we used non-parametric bootstrap with 5000 resamples (R package “boot”), reporting original OLS coefficients with 95% bias-corrected and accelerated confidence intervals and two-sided p-values.

Relationships between insomnia and depression improvements were studied using OLS regression, with bootstrap methods for BSSI outcomes.

Finally, we tested whether pre-treatment, inter-individual differences in fronto-limbic function and sleep predicted depression improvement using residualized change models [81].

## Results

### Intention-to-treat study sample

The study cohort consisted of 51 participants, recruited between May 2021 and March 2024, who completed pre-treatment timepoints (64.4% female; age 40.6 ± 10.8 years) with at least moderate depressive symptoms (Mean ± SD Pre-treatment BDI Total Score: 18.8 ± 6.4) and at least mild insomnia symptoms (Pre-treatment ISI Score: 15.6 ± 3.8). Objective sleep disturbances were confirmed by low pre-treatment sleep efficiency assessed with PSG (Pre-treatment Sleep Efficiency: 78.8% ± 15.4%). The cohort exhibited low attrition (3 of the 51 withdrawn or lost to follow-up, 5.9%). No serious adverse events were reported, and the trial phase concluded as planned. Demographic and clinical characteristics of the final sample for analysis are presented in Table 1, and the CONSORT chart (Fig. 1B) shows recruitment, enrollment and retention details. None of the primary outcomes differed by age or sex (all *p* > 0.10).

**Table 1:** Demographic sample characteristics.

Final Sample *n* = 48	%	*n*	M	SD
Sex assigned at birth (female)	66.7	32		
Gender				
Woman	64.6	31		
Man	33.3	16		
Non-binary	2.1	1		
Age (years)			40.1	10.8
Ethnicity				
Hispanic	8.3	4		
Non-Hispanic	91.7	44		
Race				
Asian	37.5	18		
Black	2.1	1		
White	52.1	25		
More than one race	4.2	2		
Prefer not to answer	4.2	2		
Marital Status				
Single	39.6	19		
Married/Partnered	45.8	22		
Divorced/Separated/Widowed	16.7	8		
Prefer not to answer	2.1	1		
Education (Years)			18.6	2.8
Employment				
Full- or part-time/student	77.1	37		
Unemployed	22.9	11		
Berlin Questionnaire (OSA Risk)				
Low Risk	54.2	26		
High Risk	35.4	17		
Apnea-Hypopnea Index (High Risk Participants)			4.8	3.12

*M* mean, *SD* standard deviation

### Treatment effects on fronto-limbic brain function

#### Amygdala reactivity to emotional faces

Consistent with the overarching hypothesis that CBT-I treatment would be associated with a reduction in fMRI-assessed limbic reactivity, participants experienced reduced amygdala reactivity following CBT-I treatment when consciously viewing fearful faces (Conscious Fear vs. Neutral Faces: *b* = –0.17 [–0.27, –0.06], p_uncorrected_ = 0.003, p_adjusted_ = 0.008, Cohen's d = 0.55; Fig. 1C and Table 2). Reduced amygdala reactivity to threat-related faces was also observed (Conscious Threat vs. Neutral Faces: *b* = –0.21 [–0.42, 0.004], P_uncorrected_ = 0.05, p_adjusted_ = 0.08, Cohen's d = 0.40) though this did not survive FDR-correction. We did not observe changes in amygdala reactivity for the nonconscious condition of the Facial Expressions of Emotion task (all p_uncorrected_ ≥ 0.88, p_adjusted_ ≥ 0.99). Similarly, there were no changes in amygdala reactivity to negative emotional images while viewing or regulating emotion in the Emotion Regulation Scenes task (all p_uncorrected_ ≥ 0.64, p_adjusted_ ≥ 0.65).

**Table 2:** Study Outcomes and Model Results.

	Pre-Treatment		Post-Treatment		LME Model Results			
	M	SD	M	SD	b (95% CI)	SE	*p*-value	Cohen’s d
Beck Depression Inventory (total minus sleep item)	17.5	6.2	10.8	8.3	–1.3 (–1.5, –1.0)	0.11	<0.0001	0.87
Beck Depression Inventory (total score)	18.9	6.5	11.5	8.7	–1.3 (–1.6, –1.1)	0.11	<0.0001	0.92
Beck Scale for Suicide Ideation	1.7	3.9	0.66	2.7	–0.29 (–0.42, –0.17)	0.07	<0.0001	0.23
Sleep Efficiency (%)	78.9	15.1	86.2	8.3	1.1 (0.42, 1.77)	0.34	0.002	0.55
Insomnia Severity Index	15.7	3.83	7.66	3.53	–1.2 (–1.33, –1.11)	0.06	<0.0001	2.02
Amygdala Activation								
Conscious Facial Expressions of Emotion Task:								
Anger > Neutral	0.11	0.36	0.05	0.26	–0.05 (–0.18, 0.08)	0.07	0.49	0.17
Fear > Neutral	0.11	0.32	–0.05	0.25	–0.17 (–0.27, –0.06)	0.05	0.003^a^	0.55
Threat > Neutral	0.22	0.60	–0.002	0.41	–0.21 (–0.42, 0.004)	0.10	0.051	0.40
Nonconscious Facial Expressions of Emotion Task:								
Anger > Neutral	0.02	0.22	0.02	0.23	0.007 (–0.08, 0.09)	0.05	0.89	0.004
Fear > Neutral	–0.01	0.24	–0.03	0.25	–0.006 (–0.10, 0.09)	0.05	0.91	0.03
Threat > Neutral	0.009	0.41	–0.01	0.41	0.001 (–0.16, 0.16)	0.08	0.99	0.02
Emotion Regulation Scenes Task:								
Negative > Neutral	0.35	0.56	0.41	0.55	0.05 (–0.16, 0.26)	0.11	0.64	0.07
Look Negative > Decrease Negative	–0.05	0.28	–0.02	0.26	0.03 (–0.08, 0.14)	0.06	0.65	0.08

*M* mean, *SD* standard deviation, *b* unstandardized coefficient, *CI* confidence interval, *SE* standard error.

Linear mixed effect (LME) model results all represent the effect of treatment with age and sex as covariates. Cohen's d values reflect pre-to-post change effect sizes (paired comparisons) and are distinct from the LME-modeled treatment effects.

^a^Significant following FDR correction (p_adjusted_ ≤ 0.05). Corrections for multiple comparisons were applied across contrasts within each task (3 contrasts for Conscious and Nonconscious tasks, 2 contrasts for the Emotion Regulation Scenes task).

### Fronto-limbic connectivity

In addition to amygdala activity, we also tested initial support for the hypothesis that insomnia treatment is associated with increased task-modulated connectivity between amygdala and five a priori subregions of the mPFC during two different emotion paradigms. Focusing on the contrast and task that demonstrated significant treatment effects, amygdala-sgACC connectivity was increased post-treatment when viewing unmasked fearful faces (Conscious Fear vs. Neutral Faces: *b* = 0.16 [0.008, 0.31], p_uncorrected_ = 0.045, p_adjusted_ = 0.23, Cohens d = 0.28; Fig. 1D).

Together, CBT-I was associated with reduced amygdala reactivity to fearful faces and a trend towards increased amygdala-sgACC connectivity that was specific to the conscious condition (Table S1 for full connectivity results).

### Treatment effects on clinical and sleep outcomes

**Fig. 2:**
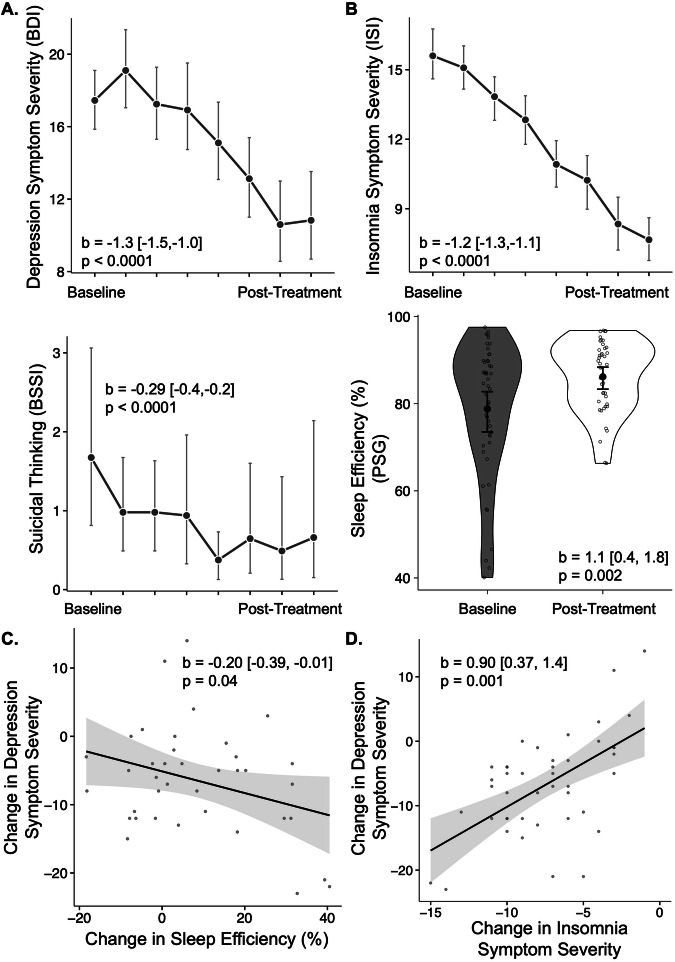
Depression and insomnia symptoms are improved following CBT-I. Lineplots show week-to-week changes in **A** depression symptom severity measured using the Beck Depression Inventory (BDI), suicidal thinking measured using the Beck Scale for Suicidal Ideation (BSSI), **B** self-reported insomnia symptom severity measured using the insomnia severity index (ISI), and sleep efficiency objectively measured using polysomnography (PSG) before and after treatment. Regression coefficients [95% CI] and p-values are from linear mixed-effect models. **C**, **D** Post- minus pre-treatment changes in objective sleep efficiency and self-reported insomnia symptoms are associated with the improvement in depressive symptoms. Regression coefficients [95% CI] and p-values are from linear regression models. The shaded areas represent 95% CI.

CBT-I was associated with improved depression and sleep outcomes. First, depression symptoms (BDI total minus sleep item) were significantly reduced over the course of treatment (*b* = –1.26 [–1.47, –1.05], *p* < 0.0001, Cohen's d = 0.87; Fig. 2A), as was the severity of suicidal thoughts (BSSI total: *b* = –0.29 [–0.42, –0.17], *p* < 0.0001, Cohens d = 0.23; Fig. 2A).

Second, participants experienced a significant increase in PSG-measured sleep efficiency (*b* = 1.1 [0.42, 1.8], *p* = 0.002, Cohens *d* = 0.54; Fig. 2B). Complementing the increase in objective sleep efficiency, participants also experienced reduced self-reported insomnia symptom severity over the course of treatment (ISI: *b* = –1.21 [–1.3, –1.1], *p* < 0.0001, Cohens d = 2.0; Fig. 2B). Thus, CBT-I treatment was associated with improved depression symptoms, as well as objective and self-reported insomnia symptoms.

### Associations between changes in depression, sleep, and fronto-limbic brain function

#### Associations between changes in depression and fronto-limbic function

We next evaluated whether changes in amygdala reactivity and its connectivity with sgACC when viewing unmasked fearful faces were associated with depression outcomes.

The treatment-related reduction in amygdala reactivity to unmasked fearful faces was not associated with the improvement in depression symptoms (*b* = –2.9 [–11.0, 5.12], *p* = 0.47, *η*^2^*p* = 0.02). Similarly, no association was found between amygdala reactivity to fearful faces and the presence of suicidal thinking (*b* = –3.4 [–12.2, 0.78], *p* = 0.48, *η*^2^*p* = 0.05). Therefore, while insomnia treatment was associated with reduced amygdala reactivity, these changes were unrelated to the parallel improvement in depression symptoms.

Next, we evaluated whether fronto-limbic connectivity, rather than activation, was associated with improved depression symptoms, again focusing on amygdala connectivity to the sgACC when viewing unmasked fearful faces. Amygdala-sgACC connectivity when viewing fearful faces was not associated with improved depression symptoms (*b* = –1.6 [–7.1, 3.8], *p* = 0.54, *η*^2^*p* = 0.01).

We repeated the above analyses for the secondary depression outcome. Decreased presence of suicidal thinking across treatment was not associated with increased amygdala-sgACC connectivity when viewing fearful faces (b = –0.08 [–2.6, 1.6], *p* = 0.92, *η*^2^*p* < 0.01).

Exploratory analyses revealed that changes in fronto-limbic activity and connectivity were also not significantly associated with improved objective sleep efficiency (see Supplement).

Taken together with the above, these initial results suggest that fronto-limbic brain connectivity may not be a mechanism of depression symptom improvement following CBT-I treatment.

### Associations between changes in depression and sleep

Subsequently, we investigated whether CBT-I-associated improvements in objective (PSG-measured sleep efficiency) and self-reported (ISI total score) insomnia symptoms were related to improvement in depression symptoms. In contrast to fronto-limbic brain function, we found that sleep outcomes were significantly associated with depression improvement. More specifically, the reduction in depression symptom severity was significantly associated with the increase in objectively measured sleep efficiency from pre- to post-treatment (*b* = –0.20 [–0.39, –0.01], *p* = 0.04, *η*^2^*p* = 0.10; Fig. 2C). A similar effect was found for the reduction in self-reported insomnia symptoms (*b* = 0.90 [0.37, 1.4], *p* = 0.001, *η*^2^*p* = 0.19; Fig. 2D). Parallel analyses using diary-derived sleep efficiency showed similar patterns (see Supplement).

These analyses were then repeated using the secondary depression outcome of suicidal thinking. The reduction in suicidal thinking was not associated with objective sleep efficiency (*b* = –0.03 [–0.18, 0.05], *p* = 0.56, *η*^2^*p* < 0.01) or with improved self-reported insomnia symptom severity (*b* = 0.35 [0.02, 0.96], *p* = 0.47, *η*^2^*p* = 0.08). Thus, depression symptom severity improvement, as assessed using the BDI after removing the sleep item, is associated with treatment-related reductions in insomnia symptoms, measured both objectively and by self-report, while more specific suicidal thinking was not.

### Moderators of depression symptom improvement

#### Pre-treatment fronto-limbic moderators

Finally, we examined whether inter-individual pre-treatment differences in fronto-limbic brain activity and connectivity, objective sleep efficiency and self-reported insomnia symptoms were predictive of the magnitude of improved clinical depression outcomes. Pre-treatment levels of amygdala reactivity and sgACC connectivity did not predict subsequent improvements in either depression or suicidal thinking (all *p* > 0.27; see Supplement for full results, which include tasks, contrasts, and mPFC target regions not engaged by CBT-I treatment).

### Pre-treatment sleep moderators

**Fig. 3:**
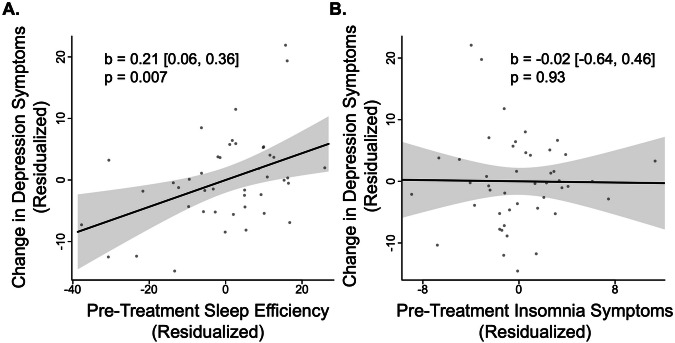
Objective sleep efficiency predicts antidepressant response following CBT-I. Added variable plots illustrating the unique relationships between the change in depression symptom severity and pre-treatment **A** objective sleep efficiency and **B** self-reported insomnia symptoms. Following a residualized change model approach, the y-axes represent post-treatment depression symptoms after accounting for baseline depression levels, age, and sex. The x-axis represents pre-treatment sleep efficiency and pre-treatment insomnia symptom severity after accounting for the same covariates. The solid line indicates the partial regression slope, with the shaded area showing the 95% CI.

In contrast, pre-treatment objective sleep efficiency, measured with PSG, was significantly predictive of the reduction in depressive symptoms (*b* = 0.21 [0.06, 0.36], *p* = 0.007, *η*^2^*p* = 0.16), such that participants with the lowest objective sleep efficiency prior to treatment experienced greater improvements in depression symptoms. On the other hand, pre-treatment self-reported insomnia symptoms, measured using the ISI questionnaire, showed no such predictive relationship (*b* = –0.02 [–0.64, 0.46], *p* = 0.93, *η*^2^*p* < 0.01; Fig. 3). This suggests that objective, but not self-reported, insomnia symptoms predict which participants will experience the greatest benefit of CBT-I for depression symptoms.

In predicting improvements in the secondary depression outcome of suicidal thinking, no such predictive relationship was found for either objective (*b* = 0.02 [–0.01, 0.13], *p* = 0.50, *η*_2_*p* < 0.01) or self-reported insomnia symptoms (*b* = –0.0006 [–0.38, 0.14], *p* = 0.99, *η*^2^*p* < 0.01).

## Discussion

In this R61 study phase, we found initial evidence that nonpharmacological insomnia treatment alters fronto-limbic emotion processing circuits. CBT-I was associated with reduced bilateral amygdala reactivity to unmasked fearful faces and marginally increased emotion-modulated amygdala-sgACC connectivity. However, there were no significant changes in fronto-limbic function during the masked (nonconscious) condition of the Facial Expressions of Emotion Task or during explicit emotion regulation conditions in the scenes task. Replicating prior work, CBT-I was associated with large within-subject improvements in depression and insomnia symptoms, including both self-reported and objectively measured sleep disturbances. Counter to our hypotheses, depression symptom improvements were not associated with changes in amygdala reactivity or amygdala-sgACC connectivity. Instead, depression improvement was associated with improved insomnia symptoms and objectively measured sleep efficiency. However, only baseline objective sleep efficiency derived from PSG, not baseline self-reported insomnia severity, predicted subsequent depression response. Collectively, these findings indicate that CBT-I-associated effects on fronto-limbic function are task-specific and dissociable from clinical depression benefits, while highlighting the importance of sleep symptoms, particularly objective sleep efficiency, as correlates and potential prognostic indicators of antidepressant response.

The first aim was to determine whether fronto-limbic circuits were modulated following CBT-I treatment in emotion reactivity and regulation task paradigms. We found that CBT-I was associated with reduced amygdala emotional reactivity and increased amygdala-sgACC connectivity for consciously presented fear-expressing faces. While most changes in amygdala reactivity and connectivity did not reach statistical significance, the observed effect sizes suggest potential treatment-related changes worth examining in larger samples. Specifically, the effect size for amygdala reactivity to consciously presented faces was moderate-large (Cohen’s d = 0.55), with moderate effect sizes for amygdala-sgACC connectivity (*d* = 0.25-0.31) and small-moderate effect sizes for amygdala-dmPFC (*d* = 0.27) and amygdala-vmPFC (*d* = 0.24) connectivity in the conscious task condition. These findings should be interpreted cautiously as preliminary evidence requiring replication. These results are broadly consistent with a normalization of the amygdala hyperreactivity and impaired prefrontal regulatory control observed in depression [32–36]. Hyperreactivity in negative affect circuits, including the amygdala, characterizes mood-congruent emotion processing bias in depression (sensitivity to negative emotion that accompanies negative mood). Previous studies also report normalization of amygdala emotional reactivity following antidepressant treatment [82–87], though not always [88, 89]. The current results are the first to demonstrate that CBT-I, a behavioral sleep intervention known to improve depression symptoms, also modifies fronto-limbic function associated with mood-congruent emotional biases and threat dysregulation.

Several factors contextualize these findings. First, reduced amygdala reactivity occurred only in the conscious condition, suggesting amygdala treatment-engagement is specific to the conscious appraisal of canonical threat cues (fearful faces) rather than automatic responses or complex scene stimuli. While previous studies found similar patterns [24, 25, 85, 90], others identified effects only for masked faces [86, 87, 91], suggesting different pathways of antidepressant effects in CBT-I compared to other treatments. Second, we observed reduced reactivity only to fear-expressing faces, overlapping with previous reports of normalization following depression treatment [20, 21, 82, 85, 90–94]. This specificity may relate to mood-congruent emotional biases in comorbid insomnia and depression, where hyperarousal and threat sensitivity [95] may produce bias towards arousing fear-expressing faces.

We did not observe changes in fronto-limbic function when explicitly regulating emotional responses in the Scenes task. Emotionally intense scenes may drive amygdala activity too strongly, overwhelming downregulation compared to face images. Alternatively, the post-treatment assessment may have been conducted too early to detect emotion regulation changes that emerge more slowly, particularly as participants continue stabilizing sleep [96]. Additionally, CBT-I’s antidepressant benefits may not operate through improved emotion regulation as measured by amygdala-mPFC function. A recent meta-analysis found emotion regulation differences in the insula and lateral PFC rather than the amygdala-mPFC regions [97]. Future work should investigate additional neurobiological mechanisms (e.g., salience network [98–100]).

Although this phase primarily targeted fronto-limbic mechanisms, the findings also support CBT-I’s clinical effects on depressive symptoms. As expected, there was a large reduction in depressive symptom severity as measured by the BDI scored without the “changes in sleeping pattern” item, indicating improvement beyond sleep complaints. Treatment was also associated with a small effect size reduction in suicidal thinking, emphasizing the clinical importance of insomnia treatment in depression, as both conditions are associated with increased suicide risk. Thus, insomnia treatment may yield broad antidepressant effects, and the second study phase will examine whether certain depression symptom profiles are more sensitive to CBT-I’s antidepressant effect.

There were large reductions in self-reported insomnia symptoms and medium increases in objective sleep efficiency. Objectively measured sleep disturbances are not always observed in insomnia disorder [101, 102] and are inconsistently affected by CBT-I [103, 104]. Our results contribute evidence that CBT-I may improve objective PSG-assessed sleep efficiency. Sleep efficiency may better correlate with subjective sleep quality [105], suggesting improvements in sleep efficiency are the objective change that patients perceive during treatment, potentially preceding downstream insomnia and mood benefits.

We hypothesized that improved depression symptoms would be associated with both fronto-limbic and sleep changes. However, despite parallel improvements, we found no associations between them, suggesting target brain mechanisms were not related to symptom improvements. This may reflect a temporal lag wherein neural changes precede depressive symptom improvements, which the single post-treatment assessment could not test. Pharmacological antidepressant treatments show similar patterns, with rapid neural changes [84] but delayed symptom effects [106]. Fronto-limbic mechanisms may be causally involved but operate early in treatment.

Changes in fronto-limbic function and symptom improvements may simply be independent, with other brain mechanisms accounting for symptom changes. Arnone et al. [82] reported a lack of correlation between depression severity and amygdala activation changes following pharmacological treatment, though the parahippocampal gyrus was related to depression severity change. Depression and insomnia are heterogeneous conditions, and different neural mechanisms may operate for different subsets of treatment response.

In contrast, depression improvements were associated with reductions in self-reported insomnia and increases in objective sleep efficiency, suggesting greater sleep benefits predicted greater antidepressant effects. Given that CBT-I specifically targets sleep, this is consistent with sleep improvements as mediating mechanisms. Few studies examined both self-reported and objective sleep in CBT-I for depression [107, 108], and many relied on actigraphy, a method with drawbacks [109]. The association with objective sleep efficiency suggests that biological pathways relating to sleep consolidation may link sleep disturbances to depression. The second phase will clarify causal roles and temporal ordering using mediation frameworks enabled by the larger sample and control treatment.

Finally, only pre-treatment objective sleep efficiency predicted the improvement in depressive symptoms, with lower pre-treatment sleep efficiency associated with greater antidepressant benefits. This finding suggests that individuals with lower objective sleep efficiency may derive greater benefit from CBT-I [110]. While the ISI is a well-validated measure of insomnia symptom severity, capturing sleep symptoms, distress, and daytime impairment, PSG-derived sleep efficiency may be more sensitive to the specific neurobiological or physiological processes that link sleep improvements to depression improvements. This preliminary finding requires replication, but if confirmed, it may have important clinical implications. While PSG is not practical for routine clinical assessment and is not part of diagnostic criteria for insomnia disorder, these findings suggest that objective sleep measures may offer distinct value beyond self-report in predicting which patients may experience the greatest antidepressant benefit from CBT-I. More accessible approaches, such as consumer-grade wearable EEG devices, could potentially serve this role, informing treatment. This is the first study examining predictors of depression response to CBT-I. Troxel et al. [111] found both objective sleep onset latency and self-reported insomnia predicted depression remission in psychotherapy/pharmacotherapy for depression, but did not examine objective sleep efficiency. The current results also suggest that predictors may differ by treatment type.

Some study limitations necessitate caution. As the first phase of a two-phase trial, the absence of a control group is a significant limitation. Without a control treatment, we cannot definitively attribute the observed improvements to CBT-I rather than natural symptom course or non-specific therapeutic factors. The two-phase clinical trial is designed to address this by including an additional active control treatment arm in the second phase, which will allow us to isolate the specific effects of CBT-I treatment. Regarding participant characteristics, while all participants experienced insomnia symptoms for at least 3 months prior to enrollment, we did not require a specific duration of depression symptoms. Additionally, the relatively small sample overrepresents females and includes a highly educated sample, potentially impacting generalizability. The sample size also limits the ability to detect smaller effects, particularly for the fronto-limbic analyses involving multiple comparisons. This may explain why several fronto-limbic associations showed moderate effect sizes but did not reach statistical significance after FDR-correction. These findings should be considered preliminary and require replication in larger samples in the next trial phase. Furthermore, while PSG is the gold-standard measure of objective sleep physiology, it is limited by the single-night collection at each time point. Adaptation effects or natural night-to-night variability may limit the representativeness of this measure, and the pre-treatment moderator findings should be interpreted as preliminary. Future studies may employ multi-night PSG or wearable EEG to address this. Finally, the a priori mechanistic brain targets do not rule out other neurobiological mechanisms.

Despite limitations, these results advance knowledge of antidepressant mechanisms of insomnia treatment for comorbid insomnia and depression. Fronto-limbic emotion processing mechanisms are engaged by treatment, but may not account for the depression response. Instead, sleep improvements may be more closely tied to depression response, with baseline sleep disturbance as a potential predictor of subsequent response.

Collectively, these findings offer initial support for a mechanism-informed framework for understanding the antidepressant benefits of insomnia treatment and reinforce the need for the randomized controlled trial design in the next phase to disentangle brain and sleep mediators and predictors of treatment response.

## Supplementary information

Table S1

Supplement

## Data Availability

The data underlying this article will be shared on reasonable request to the corresponding author.
