# Supplementary material for: Examining fronto-limbic brain and sleep mechanisms of antidepressant effects in cognitive-behavioral therapy for insomnia
Source: Neuropsychopharmacology. 2026 May 7;51(9):1657–68. doi: 10.1038/s41386-026-02431-0 (PMC13268098; doi:10.1038/s41386-026-02431-0)
Supplement: Supplementary file 2 — Supplement [file 41386_2026_2431_MOESM2_ESM.docx]

**Supplementary Materials**

**Supplementary Methods**

**Polysomnography**

PSG was used to measure sleep on the nights before the screening, pre-treatment baseline, and post-treatment timepoints using a 32-channel EEG system (Compumedics, Siesta 802) validated for sleep recording. Electroencephalography (EEG) was recorded at 26 standard locations conforming to the extended International 10-20 System (FP1, FP2, AFz F7, F3, Fz, F4, F8, FC5, FC6, C3, Cz, C4, CP5, CP6, T5, P3, Pz, P4, T6, POz, O1, O2) using an EEG cap (BrainVision Easycap). Electrooculography (EOG) was recorded at left and right outer canthi (right superior, left inferior). Electromyography (EMG) was recorded via three electrodes (one mental, two submental). Reference electrodes were recorded at both the left and right mastoid (M1, M2). Data were recorded at 512 Hz sampling rate.

Sleep staging was performed in accordance with standardized techniques^1^ using C3, C4, O1, O2, F3, F4, right and left EOG, and EMG channels. EEG and EOG were referenced to the contralateral mastoid and filtered to 0.3-35 Hz. Sleep was visually scored in 30-second epochs using the MATLAB-based Hume toolbox^2^.

**Fronto-limbic Outcomes**

Clinically-relevant circuits were generated through a meta-analytic search using Neurosynth (RRID:SCR_006798) to create a “Negative Affect” uniformity map. Regions were refined by retaining those with >50% grey matter overlap and adequate temporal signal-to-noise ratios. From this refined set, five regions of the mPFC were selected for the current analyses. This included the dorsal anterior cingulate cortex (dACC; MNI center of mass: 3.3, 21.7, 33.3; Num. voxels: Mean±SD = 246.8±12.7, Range = 206-269; Volume: 1974.5±101.3mm^3^**),** ventromedial prefrontal cortex (vmPFC; MNI center of mass: -2.3, 42.1, -15.3; Num. voxels: Mean±SD = 68.5±6.0, Range = 50-76; Volume: 547.9±47.8mm^3^)**,** dorsomedial prefrontal cortex (dmPFC; MNI center of mass: 3.7, 4.3, 55.4; Num. voxels: Mean±SD = 143.6±13.2, Range = 112-163; Volume: 1149.1±105.7mm^3^), subgenual anterior cingulate cortex (sgACC; MNI center of mass: 2.7m, 22.8, -9.0; Num. voxels: Mean±SD = 64.8±3.1, Range = 58-71; Volume: 518.4±24.4mm^3^), pregenual anterior cingulate cortex (pACC; MNI center of mass: -4.7, 43.5, -3.8; Num. voxels: Mean±SD = 128.1±5.1, Range = 113-135; Volume: 1024.5±40.8mm^3^)**.** The sgACC did not meet quality control metrics for temporal signal-to-noise ratio, but given the difficulty of imaging this region, its importance to defining the negative affect circuit, and prior imaging findings in depression, we included the region in the current analyses. For subcortical amygdala, an anatomical definition from the AAL atlas was used.

**Imaging Tasks**

There were two conditions of the Facial Expressions of Emotion Task: a conscious condition in which each emotional facial expression was presented for 500ms, with an interstimulus interval of 750ms, and a nonconscious condition in which stimuli were presented for 16.7ms immediately followed by a neutral face mask to prevent conscious processing, with an interstimulus interval of 1083ms to align with the conscious condition. Stimuli were drawn from a standardized series of facial expressions of threat-related emotions (fear, anger), loss-related emotions (sadness), and reward-related emotions (happiness), along with neutral faces, and modified to have equivalent luminance and to align the eye level in the center of each image. In both conditions, a total of 240 stimuli were grouped in blocks of 8 faces of the same emotion depicted by different individuals, with each emotion block repeated 5 times and presented in a pseudorandom order. In the nonconscious condition, mask stimuli were offset slightly by 1° in each diagonal direction to control for the possibility that participants can nonconsciously detect emotions due to different flicker effects in the brief transition from emotional expression to neutral mask stimulus. To promote active attention to the task, participants were instructed to actively attend in order to answer post-scan questions about the face stimuli.

In the Emotion Regulation Scenes Task, trials began with a 2 second cue prompting participants to either “look” or “decrease” their emotional response to the subsequent emotional image. Either a negative or neutral image was then presented for 7 seconds, followed by a 4 second period when they are asked to rate their level of emotional negativity on a scale from 1 (Not at all negative) to 5 (Very much negative). A 1-3 second variable duration fixation screen preceded the onset of the next trial. Images were presented in a pseudorandom order such that no more than two of the same instructions or 4 negative stimuli could be presented consecutively. Of the negative images, 15 were paired with the instruction “look” and 15 were paired with the instruction “decrease”. All 15 neutral images were presented with the instruction “look”. Prior to entering the scanner, participants were given instructions on strategies for both the “look” and “decrease” conditions and practiced with several images that were not repeated during scan acquisition.

**MRI Acquisition**

During each of the above fMRI tasks, blood oxygenation level-dependent (BOLD) contrast functional images were acquired with echo-planar imaging (EPI) MR sequences on a 3.0T GE Discovery MR750 scanner (GE Healthcare, Milwaukee, Wisconsin) with a 32-channel head coil with the following parameters: TR=2000ms, TE=27.5ms, matrix=64x64, FOV=24cm, flip angle=77°. Each whole brain volume included 45 interleaved 3mm thick axial slices. A 3D SPGR sequence was collected for normalization of functional images to standard space, as well as a field map for EPI unwarping. For the Facial Expression of Emotion Task, 154 volumes were collected over 5min and 8s for both the conscious and nonconscious conditions**.** For the Emotion Regulation Scenes Task, 400 volumes were acquired over 13min and 20s. To ensure BOLD saturation, three dummy volumes were included at the start of each run. A high-resolution T1-weighted structural was acquired using an HCP-equivalent inversion recovery fast SPGR sequence with an inversion time of 450ms, a TR of 6.3ms, a TE of 2.8ms, and a flip angle of 12°. The field of view was 24cm with a 320x320 matrix size and a slice thickness of 0.8mm (isotropic voxel size 0.8mm^3^).

**MRI Analysis**

Preprocessing of fMRI data was performed using *fMRIPrep 22.1.1*^3^ (RRID:SCR_016216) which is based on *Nipype 1.8.5*^4,5^ (RRID:SCR_002502) and FSL^6^ (6.0.5.1:57b01774). T1w anatomical scans were normalized to standard space of the Montreal Neurological Institute (MNI152) template using FSL nonlinear registration FLIRT and FNIRT (FSL). For each BOLD run, the following preprocessing was performed. First, a reference volume and its skull-stripped version were generated using a custom methodology of *fMRIPrep*. Head-motion parameters with respect to the BOLD reference (transformation matrices and six corresponding rotation and translation parameters) were estimated before any spatiotemporal filtering using cmflirt (FSL). The estimated fieldmap was then aligned with rigid-registration to the target EPI reference run. The field coefficients were mapped on to the reference EPI using the transform. BOLD runs were slice-time corrected to 0.978s (0.5 of slice acquisition range of 0s-1.96s) using 3dTshift from AFNI^7^ (RRID:SCR_005927). The BOLD reference was then co-registered to the T1w reference using bbregister (FreeSurfer) which implements boundary-based registration^8^. Co-registration was configured with six degrees of freedom. Several confounding time-series were calculated based on the preprocessed BOLD: framewise displacement (FD), DVARS and three region-wise global signals. FD was computed using two formulations following Power^9^ (absolute sum of relative motions) and Jenkinson et al.^6^. FD and DVARS were calculated for each functional run, both using their implementations in *Nipype* (following the definitions by Power). The three global signals were extracted within cerebrospinal fluid, white matter, and whole-brain masks. The head-motion estimates calculated in the correction step were also used for confound correction in the models. The confound time series derived from head motion estimates and global signals was also expanded with the inclusion of temporal derivatives^10^. Frames that exceed a threshold of 0.5mm FD or 1.5 standardized DVARS were annotated as motion outliers, as were the immediately previous and subsequent two volumes. The BOLD time-series was resampled into standard space, generating a preprocessed BOLD run in MNI152NLin2009cAsym space. Finally, spatial smoothing was performed with an isotropic, Gaussian kernel of 6mm full-width half-maximum.

For each participant, trial-related activity was assessed by convolving a vector of trial/block onsets with a canonical hemodynamic response function in the context of a GLM using SPM12 (https://www.fil.ion.ucl.ac.uk/spm/) in each task. Twelve movement-related covariates from realignment (three rigid-body translations and three rotations and their first temporal derivatives), along with global time-series from cerebrospinal fluid and white matter masks were used as regressors of no-interest in the design matrix. In addition, temporal masks were created for outlier volumes (volumes exceeding 0.5mm FD or 1.5 standardized DVARS and the immediately preceding and subsequent two volumes) and used as regressors of no interest in the first-level statistical models. Connectivity between the mPFC and amygdala was assessed using PPI analyses^11^ with the bilateral amygdala as the seed region. Beta values for each participant and ROI were extracted for statistical analyses.

**Sleep Diaries**

A single-night measurement of sleep using PSG only provides a snapshot of sleep pre-/post-CBT-I treatment. Thus, we also examined sleep efficiency as self-reported in sleep diaries. For each participant, we defined two analysis windows based on the timing of CBT-I treatment in the study timeline. First, a pre-treatment period was defined as all sleep diaries prior to the first CBT-I treatment session, and a post-treatment period was defined as all diaries prior to the end of study participation and after the final CBT-I treatment session. For each sleep diary, we calculated sleep efficiency and total sleep duration, based on participant reports. First, total sleep time was calculated as total dark time (the total sleep opportunity) minus sleep onset latency, wake after sleep onset, and early awakening duration. Then, sleep efficiency was calculated by taking the ratio of total sleep time to total dark time and multiplying by 100. Next, for analyses examining diary-derived sleep parameters around major assessment timepoints (e.g. PSG recordings and fMRI scanning), we selected up to 6 nights of diary entries anchored to each PSG recording night but not including the PSG night. For pre-treatment diaries, if fewer than 6 pre-PSG diaries were available, diaries following the PSG night but prior to the first CBT-I treatment session were included. The average total sleep time (TST, minutes) and sleep efficiency (%) were calculated by averaging across the 6 nights pre-treatment and post-treatment diaries, respectively.

**Supplementary Results**

**Fronto-limbic Connectivity Full Results**

| Conscious Facial Expressions of Emotion Task | | | | | | | | | |
| --- | --- | --- | --- | --- | --- | --- | --- | --- | --- |
|  |  | Pre-Treatment | | Post-Treatment | | LME Model Results | | | |
| Target - Seed | Contrast | M | SD | M | SD | b (95% CI) | SE | p_unc_ | Cohen’s d |
| dACC - Amygdala | Anger > Neutral | -0.10 | 0.49 | -0.07 | 0.50 | 0.03 (-0.16, 0.23) | 0.10 | 0.75 | 0.03 |
|  | Fear > Neutral | 0.004 | 0.49 | 0.03 | 0.47 | 0.02 (-0.17, 0.21) | 0.10 | 0.85 | 0.02 |
|  | Threat > Neutral | -0.02 | 0.28 | 0.003 | 0.27 | 0.02 (-0.10, 0.14) | 0.06 | 0.73 | 0.03 |
| dmPFC - Amygdala | Anger > Neutral | -0.09 | 0.46 | -0.20 | 0.40 | -0.11 (-0.25, 0.02) | 0.07 | 0.11 | 0.27 |
|  | Fear > Neutral | -0.04 | 0.46 | -0.05 | 0.42 | -0.02 (-0.20, 0.17) | 0.10 | 0.86 | 0.03 |
|  | Threat > Neutral | -0.03 | 0.28 | -0.06 | 0.23 | -0.03 (-0.14, 0.07) | 0.05 | 0.51 | 0.12 |
| pACC - Amygdala | Anger > Neutral | 0.03 | 0.45 | -0.03 | 0.43. | -0.05 (-0.23, 0.14) | 0.09 | 0.60 | 0.10 |
|  | Fear > Neutral | 0.007 | 0.55 | -0.06 | 0.43 | -0.06 (-0.24, 0.12) | 0.09 | 0.50 | 0.17 |
|  | Threat > Neutral | 0.006 | 0.26 | -0.04 | 0.23 | -0.04 (-0.14, 0.07) | 0.05 | 0.48 | 0.16 |
| sgACC - Amygdala | Anger > Neutral | -0.08 | 0.35 | 0.06 | 0.47 | 0.12 (-0.05, 0.29) | 0.09 | 0.17 | 0.25 |
|  | Fear > Neutral | -0.09 | 0.39 | 0.08 | 0.51 | 0.16 (0.01, 0.31 | 0.08 | 0.04 | 0.28 |
|  | Threat > Neutral | -0.05 | 0.20 | 0.05 | 0.28 | 0.10 (0.01, 0.18) | 0.04 | 0.04 | 0.31 |
| vmPFC - Amygdala | Anger > Neutral | -0.002 | 0.44 | -0.15 | 0.44 | -0.14 (-0.33, 0.04) | 0.09 | 0.13 | 0.24 |
|  | Fear > Neutral | -0.08 | 0.51 | -0.14 | 0.48 | -0.07 (-0.24, 0.10) | 0.09 | 0.45 | 0.19 |
|  | Threat > Neutral | -0.03 | 0.24 | -0.09 | 0.26 | -0.05 (-0.16, 0.05) | 0.05 | 0.30 | 0.23 |
|  |  |  |  |  |  |  |  |  |  |
| Nonconscious Facial Expressions of Emotion Task | | | | | | | | | |
|  |  | Pre-Treatment | | Post-Treatment | | LME Model Results | | | |
| Target - Seed | Contrast | M | SD | M | SD | b (95% CI) | SE | p_unc_ | Cohen’s d |
| dACC - Amygdala | Anger > Neutral | 0.06 | 0.45 | -0.09 | 0.54 | -0.13 (-0.29, 0.04) | 0.09 | 0.15 | 0.19 |
|  | Fear > Neutral | -0.04 | 0.42 | -0.08 | 0.56 | -0.03 (-0.21, 0.16) | 0.09 | 0.78 | 0.06 |
|  | Threat > Neutral | 0.02 | 0.26 | -0.04 | 0.32 | -0.04 (-0.15, 0.07) | 0.05 | 0.45 | 0.10 |
| dmPFC - Amygdala | Anger > Neutral | -0.007 | 0.50 | -0.02 | 0.44 | -0.01 (-0.20, 0.18) | 0.10 | 0.91 | 0.02 |
|  | Fear > Neutral | -0.04 | 0.40 | -0.05 | 0.42 | -0.01 (-0.18, 0.16) | 0.09 | 0.91 | 0.04 |
|  | Threat > Neutral | 0.02 | 0.27 | 0.006 | 0.23 | -0.01 (-0.12, 0.09) | 0.05 | 0.84 | 0.02 |
| pACC - Amygdala | Anger > Neutral | -0.06 | 0.50 | -0.08 | 0.47 | -0.04 (-0.21, 0.13) | 0.09 | 0.67 | 0.08 |
|  | Fear > Neutral | -0.09 | 0.41 | -0.06 | 0.56 | 0.02 (-0.17, 0.21) | 0.10 | 0.82 | 0.07 |
|  | Threat > Neutral | -0.04 | 0.30 | -0.03 | 0.29 | 0.0006 (-0.10, 0.11) | 0.05 | 0.99 | 0.002 |
| sgACC - Amygdala | Anger > Neutral | -0.10 | 0.46 | 0.005 | 0.37 | 0.09 (-0.07, 0.24) | 0.08 | 0.27 | 0.23 |
|  | Fear > Neutral | -0.0001 | 0.40 | -0.004 | 0.44 | -0.01 (-0.18, 0.15) | 0.09 | 0.88 | 0.03 |
|  | Threat > Neutral | -0.03 | 0.30 | -0.006 | 0.22 | 0.01 (-0.09, 0.11) | 0.05 | 0.85 | 0.09 |
| vmPFC - Amygdala | Anger > Neutral | -0.03 | 0.48 | 0.01 | 0.53 | 0.03 (-0.18, 0.23) | 0.11 | 0.79 | 0.03 |
|  | Fear > Neutral | 0.04 | 0.49 | 0.03 | 0.54 | -0.02 (-0.23, 0.18) | 0.10 | 0.82 | 0.02 |
|  | Threat > Neutral | 0.01 | 0.30 | 0.03 | 0.28 | 0.004 (-0.11, 0.11) | 0.06 | 0.95 | 0.01 |
|  |  |  |  |  |  |  |  |  |  |
| Emotion Regulation Scenes Task | | | | | | | | | |
|  |  | Pre-Treatment | | Post-Treatment | | LME Model Results | | | |
| Target - Seed | Contrast | M | SD | M | SD | b (95% CI) | SE | p_unc_ | Cohen’s d |
| dACC - Amygdala | Negative > Neutral | 0.05 | 0.24 | 0.08 | 0.39 | 0.03 (-0.10, 0.17) | 0.07 | 0.64 | 0.08 |
|  | Look Negative > Decrease Negative | -0.02 | 0.49 | 0.08 | 0.34 | 0.12 (-0.05, 0.30) | 0.09 | 0.18 | 0.13 |
| dmPFC - Amygdala | Negative > Neutral | 0.03 | 0.23 | 0.07 | 0.29 | 0.029 (-0.08, 0.13) | 0.05 | 0.59 | 0.12 |
|  | Look Negative > Decrease Negative | -0.04 | 0.45 | 0.01 | 0.44 | 0.08 (-0.11, 0.26) | 0.10 | 0.43 | 0.05 |
| pACC - Amygdala | Negative > Neutral | -0.03 | 0.24 | -0.02 | 0.33 | 0.01 (-0.10, 0.13) | 0.06 | 0.83 | 0.06 |
|  | Look Negative > Decrease Negative | 0.06 | 0.40 | 0.10 | 0.37 | 0.03 (-0.13, 0.18) | 0.08 | 0.73 | 0.05 |
| sgACC - Amygdala | Negative > Neutral | -0.03 | 0.23 | 0.02 | 0.26 | 0.03 (-0.09, 0.14) | 0.06 | 0.66 | 0.18 |
|  | Look Negative > Decrease Negative | 0.06 | 0.41 | 0.07 | 0.47 | 0.002 (-0.18, 0.19) | 0.09 | 0.99 | 0.01 |
| vmPFC - Amygdala | Negative > Neutral | 0.02 | 0.25 | 0.03 | 0.30 | -0.003 (-0.12, 0.11) | 0.06 | 0.96 | 0.04 |
|  | Look Negative > Decrease Negative | 0.05 | 0.44 | 0.10 | 0.44 | 0.03 (-0.12, 0.19) | 0.08 | 0.69 | 0.09 |

**Table S1.** Linear mixed effect (LME) model results all represent the effect of treatment with age and sex as covariates. Cohens d values reflect pre-to-post change effect sizes (paired comparisons) and are distinct from the LME-modeled treatment effects. Abbreviations: M=mean, SD=standard deviation, b=unstandardized coefficient, CI=confidence interval, SE=standard error, dACC=dorsal anterior cingulate cortex, dmPFC=dorsomedial prefrontal cortex, pACC=pregenual anterior cingulate cortex, sgACC=subgenual anterior cingulate cortex, vmPFC=ventromedial prefrontal cortex.

***** Significant following FDR correction (p_adjusted_ ≤ 0.05). Correction for multiple comparisons was applied across PPI models for each task.

**Associations Between Changes in Depression and Fronto-limbic Function**

Exploratory analyses evaluated associations between fronto-limbic activation and connectivity changes with depressive symptom improvements for all remaining tasks, contrasts, and mPFC subregions that did not demonstrate significant modification following CBT-I treatment. As with the reduced amygdala reactivity to unmasked fear-expressing faces, there was no significant association with improved depressive symptom severity for all tasks and contrasts (Conscious Faces: all p_uncorrected_ ≥ 0.53, p_adjusted_ ≥ 0.69; Nonconscious Faces: all p_uncorrected_ ≥ 0.41, p_adjusted_ ≥ 0.84; Emotion Regulation Scenes task: all p_uncorrected_ ≥ 0.88, p_adjusted_ ≥ 0.91). A similar lack of association was found between amygdala reactivity and the presence of suicidal thinking across all three tasks (Conscious Faces: all p_uncorrected_ ≥ 0.48, p_adjusted_ ≥ 0.71; Nonconscious Faces: all p_uncorrected_ ≥ 0.51, p_adjusted_ ≥ 0.54; Emotion Regulation Scenes: all p_uncorrected_ ≥ 0.51, p_adjusted_ ≥ 0.87). Therefore, while insomnia treatment was associated with reduced amygdala reactivity, these limbic brain changes were unrelated to the parallel improvement in depression symptoms.

We evaluated the same associations for amygdala-mPFC connectivity. There were also no associations between depression improvement and amygdala-sgACC connectivity when viewing threatening or angry faces in the same conscious task condition (all p_uncorrected_ ≥ 0.53, p_adjusted_ = 0.99). We then tested whether amygdala-mPFC connectivity across all three tasks and regions of interest was associated with improved depression symptoms following treatment. No mPFC region’s connectivity to the amygdala was significantly related to depression symptom improvement for both the conscious (all p_uncorrected_ ≥ 0.15, p_adjusted_ ≥ 0.97) and nonconscious (all p_uncorrected_ ≥ 0.16, p_adjusted_ ≥ 0.75) versions of the Facial Expressions of Emotion Task. There was a trending association for the Emotion Regulation Scenes task between increased amygdala-vmPFC connectivity when viewing negative scenes and depression symptom improvement (b = -5.6 [-11.9, 0.72], p_uncorrected_ = 0.08, p_adjusted_ = 0.59). Decreased presence of suicidal thinking across treatment was not significantly associated with fronto-limbic connectivity for all tasks following FDR correction (Conscious Faces: all p_uncorrected_ ≥ 0.48, p_adjusted_ ≥ 0.89; Nonconscious Faces: all p_uncorrected_ ≥ 0.46, p_adjusted_ ≥ 0.85; Emotion Regulation Scenes: all p_uncorrected_ ≥ 0.48, p_adjusted_ = 0.80).

**Moderators of Depression Symptom Improvement**

**Pre-treatment Fronto-limbic Moderators**

We performed additional exploratory analyses on fronto-limbic brain targets that did not demonstrate significant modifications following treatment, here testing pre-treatment associations with depressive symptom improvement. Similar null results for pre-treatment amygdala reactivity from the conscious condition of the Facial Expressions of Emotion Task predicting depression symptom improvement were also observed for threatening and angry faces (all: p_uncorrected_ ≥ 0.82, p_adjusted_ ≥ 0.95).

We observed a significant association between lower pre-treatment amygdala reactivity to angry faces, this time under nonconscious face stimuli conditions, with improved depressive symptoms (b = 18.3 [8.9, 27.6], p_uncorrected_ = 0.0003, p_adjusted_ = 0.001). This result aligns with previous findings of pharmacological treatment of depression^12^. There were no significant associations found for fearful or threatening faces in the nonconscious condition (all: p_uncorrected_ ≥ 0.17, p_adjusted_ ≥ 0.33). There were also no significant associations for the Emotion Regulation Scenes task (all: p_uncorrected_ ≥ 0.36, p_adjusted_ ≥ 0.62).

Pre-treatment amygdala emotional face reactivity was not predictive of improvements in suicidal thinking across all tasks (Conscious Faces: all p_uncorrected_ ≥ 0.57, p_adjusted_ ≥ 0.61; Nonconscious Faces: all p_uncorrected_ ≥ 0.46, p_adjusted_ ≥ 0.49; Emotion Regulation Scenes: all p_uncorrected_ ≥ 0.47, p_adjusted_ = 0.93).

Again, similar lack of significant associations were found for angry and threatening faces from the same task, this time looking at mPFC connectivity (all: p_uncorrected_ ≥ 0.09, p_adjusted_ ≥ 0.64. From the nonconscious condition of the Facial Expressions of Emotion Task, however, task-modulated connectivity between the amygdala and sgACC when viewing fearful faces was marginally associated with improved depression symptoms (b = 5.5 [-0.15, 11.1], p_uncorrected_ = 0.06, p_adjusted_  = 0.58).

From the Emotion Regulation Scenes task, we found that pre-treatment connectivity between the amygdala and both the dACC and dmPFC when viewing negative emotional scenes was associated with the improvement in depression symptoms, such that participants with lower pre-treatment connectivity experienced a relatively larger decrease in depression symptoms (dACC: b = 11.8 [2.8, 20.7], p_uncorrected_ = 0.01, p_adjusted_ = 0.10; dmPFC: b = 11.8 [2.0, 21.6], p_uncorrected_ = 0.02, p_adjusted_ = 0.10).

Similarly, pre-treatment task-modulated amygdala-mPFC connectivity was not significantly predictive of the reductions in suicidal thinking for all three tasks and mPFC target regions (Conscious Faces: all p_uncorrected_ ≥ 0.44, p_adjusted_ ≥ 0.93; Nonconscious Faces: all p_uncorrected_ ≥ 0.44, p_adjusted_ ≥ 0.59; Emotion Regulation Scenes: all p_uncorrected_ ≥ 0.43, p_adjusted_ = 0.56).

**Associations Between Diary-derived Sleep Efficiency and Primary Outcomes**

We replicated the primary analyses which included PSG-derived sleep efficiency using diary-derived sleep efficiency, with comparable results. First, we specified a linear mixed effect model to test the effect of treatment on diary-derived sleep efficiency from the recent sleep history prior to each timepoint. This showed significant effects of treatment, similar to the objective PSG data (b=1.2 [0.7, 1.7], p<0.0001). Next, we tested whether treatment-related changes in diary-derived sleep efficiency also were associated with improvements in depressive symptoms. While both objective and self-reported sleep efficiency were similarly improved following treatment, only the change in objective sleep efficiency showed a statistically significant association with the reduction in depressive symptoms (b=-0.16 [-0.35, 0.03], p=0.10), though they are directionally comparable.

Similar to the objective sleep efficiency measure, improved diary-derived sleep efficiency was not significantly associated with changes in either amygdala reactivity or amygdala-mPFC connectivity (all p_uncorrected_>0.26, p_corrected_>0.93).

Last, we found that pre-treatment sleep efficiency derived from diaries predicted the reduction in depressive symptoms following treatment, though at a trend level of significance (b=0.19 [-0.004, 0.4], p=0.055).

Taken together, sleep efficiency, whether derived from objective PSG or self-reported sleep diaries, is improved by CBT-I and associated with depressive symptoms, but PSG-derived sleep efficiency may be more closely associated, possibly because it is an objective brain-based measure of sleep.

**Associations Between PSG-derived Sleep Efficiency and Fronto-limbic Outcomes**

We conducted exploratory analyses examining whether changes in PSG-derived sleep efficiency are associated with changes in fronto-limbic functioning. We found that the improvement in objective sleep efficiency was not significantly associated with reduced amygdala reactivity (Conscious Fear vs Neutral Faces: b=0.0007 [-0.009, 0.01], p_uncorrected_=0.89). Similar lack of significant associations was observed for amygdala-mPFC connectivity (all p_uncorrected_>0.14, p_corrected_>0.98).

**Associations Between Diary-derived Total Sleep Time and Fronto-limbic Outcomes**

We analyzed the associations between changes in total sleep time derived from (1) PSG and (2) sleep diaries, with similar null results for both, matching the primary results for objective sleep efficiency. For changes in PSG total sleep time, there were no significant associations with either amygdala reactivity (Conscious Fear vs Neutral Faces Amygdala reactivity: b=0.005 [-0.001, 0.001], p_uncorrected_=0.94) or connectivity (Conscious Fear vs Neutral Faces Amygdala-mPFC connectivity: all p_uncorrected_>0.07, p_corrected_≥0.79) changes. Similar null results were found for diary-derived total sleep time for both amygdala reactivity (Conscious Fear vs Neutral Faces Amygdala reactivity: b=0.0002 [-0.002, 0.002], p_uncorrected_=0.82) and connectivity (Conscious Fear vs Neutral Amygdala-mPFC connectivity: all p_uncorrected_>0.27, p_corrected_≥0.94).

**Associations Between Changes in Depression and Amygdala Reactivity and Sleep**

We repeated the current analyses examining associations between the improved depressive symptoms and reduced amygdala reactivity, increased sleep efficiency, and reduced insomnia symptoms using an alternative modeling approach applying residualized change models. In these models, pre-treatment starting points for depressive symptoms are explicitly included, accounting for individual differences prior to treatment. This alternative approach produced comparable results. While accounting for pre-treatment depressive symptom severity, there was no significant association between the reduction in amygdala reactivity and improved depressive symptoms (Conscious Fear vs. Neutral Faces: b=-4.2 [-12.1, 3.7], p_uncorrected_=0.29). Similar results were found for improved sleep efficiency (b=-0.19 [-0.38, -0.004], p=0.0004) and reduced insomnia symptoms (b=0.96 [0.45, 1.5], p=0.0005) in predicting the reduced depressive symptoms.

**References**

1. Berry, R. B. *et al.* AASM Scoring Manual Updates for 2017 (Version 2.4). *J Clin Sleep Med* **13**, 665–666 (2017).

2. Saletin, J. & Greer, S. Hume Software Package (previously sleepSMG): Open‐Source MATLAB User Interface for Scoring Sleep.

3. Esteban, O. *et al.* fMRIPrep: a robust preprocessing pipeline for functional MRI. *Nat Methods* **16**, 111–116 (2019).

4. Gorgolewski, K. *et al.* Nipype: a flexible, lightweight and extensible neuroimaging data processing framework in python. *Front Neuroinform* **5**, 13 (2011).

5. Gorgolewski, K. J. *et al.* The brain imaging data structure, a format for organizing and describing outputs of neuroimaging experiments. *Sci Data* **3**, 160044 (2016).

6. Jenkinson, M., Bannister, P., Brady, M. & Smith, S. Improved optimization for the robust and accurate linear registration and motion correction of brain images. *Neuroimage* **17**, 825–841 (2002).

7. Cox, R. W. & Hyde, J. S. Software tools for analysis and visualization of fMRI data. *NMR Biomed* **10**, 171–178 (1997).

8. Greve, D. N. & Fischl, B. Accurate and robust brain image alignment using boundary-based registration. *Neuroimage* **48**, 63–72 (2009).

9. Power, J. D. *et al.* Methods to detect, characterize, and remove motion artifact in resting state fMRI. *Neuroimage* **84**, 320–341 (2014).

10. Satterthwaite, T. D. *et al.* An improved framework for confound regression and filtering for control of motion artifact in the preprocessing of resting-state functional connectivity data. *Neuroimage* **64**, 240–256 (2013).

11. McLaren, D. G., Ries, M. L., Xu, G. & Johnson, S. C. A Generalized Form of Context-Dependent Psychophysiological Interactions (gPPI): A Comparison to Standard Approaches. *Neuroimage* **61**, 1277–1286 (2012).

12. Williams, L. M. *et al.* Amygdala Reactivity to Emotional Faces in the Prediction of General and Medication-Specific Responses to Antidepressant Treatment in the Randomized iSPOT-D Trial. *Neuropsychopharmacology* **40**, 2398–2408 (2015).
